# Life span pigmentation changes of the substantia nigra detected by neuromelanin‐sensitive MRI

**DOI:** 10.1002/mds.27502

**Published:** 2018-11-13

**Authors:** Yue Xing, Abdul Sapuan, Rob A. Dineen, Dorothee P. Auer

**Affiliations:** ^1^ Radiological Sciences, Division of Clinical Neuroscience School of Medicine, University of Nottingham Nottingham UK; ^2^ Sir Peter Mansfield Imaging Centre University of Nottingham Nottingham UK; ^3^ Nottingham NIHR Biomedical Research Centre Nottingham UK

## Abstract

**Background**: Neuromelanin is a pigment with strong iron‐chelating properties preferentially found in dopaminergic neurons of the substantia nigra pars compacta (SNpc). Parkinson's disease is characterized by pronounced, MRI‐detectable neuromelanin loss, but the neuroprotective or neurotoxic role of neuromelanin remains debated. Histological studies have demonstrated neuromelanin increases with age, but this has not been confirmed in vivo, and there is uncertainty whether neuromelanin declines, stabilizes, or increases from middle age.

**Methods**: This study aimed to establish physiological changes of pigmentation of the SNpc using a pooled data set of neuromelanin‐sensitive 3T MRI from 134 healthy individuals aged 5‐83 years. Neuromelanin‐related brightness (regional contrast to ratio) and calibrated hyperintense volumes were analyzed using linear and nonlinear regression models to characterize age effects. Laterality, sex, and subregional effects were also assessed.

**Results**: For brightness, age effects were best described as a quadratic trajectory explaining 81.5% of the observed variance in the SNpc showing a strong increase from childhood to adolescence, with plateauing in middle age and a decline in older age. Similar but less pronounced effects were seen in hyperintense volumes. We also show an anterior‐posterior gradient in SNpc contrast, larger normalized neuromelanin‐rich volume in women > 47 years old, but no laterality effect.

**Conclusions**: Using optimized neuromelanin MRI in a life span sample, we demonstrate a strong age effect with inverted U‐shaped SNpc pigmentation‐related contrast from childhood to old age. This age trajectory of physiological SNpc pigmentation needs to be taken into account for diagnostic applications of depigmentation. The study also paves the way for systematic investigations of the mechanisms of neuromelanin in healthy and pathological brain development and aging. © 2018 The Authors. Movement Disorders published by Wiley Periodicals, Inc. on behalf of International Parkinson and Movement Disorder Society.

Neuromelanin (NM) is a dark pigmented granule preferentially found in catecholaminergic neurons, such as the substantia nigra (SN) and locus coeruleus (LC). NM acts as a scavenger that removes excess potentially toxic substances through the autooxidation of catecholamines and/or binding redox‐active metal ions.^1^ Given this capacity to bind toxins and in particular ferric iron, NM may be neuroprotective,^2^ but when released from degenerating neurons, it may also induce immune‐based pathogenic mechanisms and trigger neurodegeneration.^3^, ^4^

Parkinson's disease (PD) has been hypothesized to result from accelerated aging because of exacerbation of oxidative stress in the SN in addition to environmental toxins in which NM plays an essential and catalytic role.^5^ Neuromelanin chelated with ferric iron has paramagnetic properties with subsequent T1 shortening.^6^, ^7^ High‐resolution short–echo time T1‐weighted MRI scans with additional magnetization transfer contrast allows reliable and sensitive detection of paramagnetic neuromelanin in the SN pars compacta and locus coeruleus.^8^, ^9^, ^10^ The corresponding T1 hyperintensity is considered to reflect the amount of ferric iron bound to NM.^11^, ^12^ In the context of PD and other neurodegenerative diseases, previous NM imaging has consistently reported a reduction of T1 hyperintensity in the SN and LC, and a link between symptom severity and quantitative measures of neuromelanin loss.^8^, ^13^, ^14^, ^15^, ^16^, ^17^ Robust normalization and quantification procedures were developed for scanner‐ and protocol‐independent data analysis as a prerequisite for multicenter trials and clinical applications.^16^, ^17^, ^18^ One potential limitation of these studies is the lack of detailed understanding of the physiological variation of SN pigmentation in vivo across the life span. Previous histological studies found pigmented granules in human SN at around the age of 2‐3 years, with accumulation as age increased.^19^ The size and number of granules remained stable after the second decade of life,^20^ whereas other studies reported an age‐dependent loss^21^, ^22^, ^23^ or a continued increase.^24^ The preclinical evidence based on animal studies is very limited because NM is hardly expressed in nonprimate brains.^25^ Using NM‐sensitive MRI, an early in vivo MRI study suggested a nonlinear age effect for human LC.^26^ However, in vivo characterization of the physiological changes of the NM‐related signal in the SN and its subregions during brain development and age‐related degeneration is lacking. This study aimed to investigate age‐related NM signal variation in the SN as well as other physiological factors using optimized neuromelanin‐sensitive MRI in a comprehensive life span sample.

## Material and Methods

### Participants

This retrospective study pooled data of healthy participants across several prospectively recruiting studies with Research Ethics Committee approval conducted within our institution, as detailed in the supplementary material. All participants aged 16 years and older gave informed consent, and consent was provided by the parent or guardian for participants younger than 16 years. Data were collected from February 2015 to May 2018, and 134 healthy control volunteers who were free from major neurologic, psychiatric, or medical disorders were included (for details, see the inclusion and exclusion criteria in the supplementary Materials and Methods).

### Imaging Acquisition

All participants underwent MRI using a 3T GE scanner (Discovery MR750; GE Healthcare, Milwaukee, WI) with a 32‐channel head coil. The head was stabilized with inflated foam padding. First, conventional sagittal T1 images were obtained for setting up the scanning field. The 30‐dimnesional spoiled gradient recalled neuromelanin‐sensitive T1‐weighted images with magnetization transfer were acquired with the following parameters that were optimized on the basis of a previous protocol^27^: TR, 38.4 milliseconds; TE, 3 milliseconds; flip angle, 20; slice thickness, 2 mm; Field of view (FOV), 19.2; matrix: 480 × 192; scanning time, 3.25 minutes. Thirty axial image slices were obtained on each individual with parallel alignment to the anterior callosum‐posterior callosum line.

### Image Processing

We first chose the 3 slices that contained substantia nigra by first identifying the red nuclei as landmarks and then chose the 3 slices below that slice. In‐house Matlab‐based semiautomated approaches were used to compute (1) the contrast‐to‐background ratio (CBR) and (2) the suprathreshold hyperintense volume of the neuromelanin‐rich region (a fully manual version of the same method is described in reference 17, and the delineation of ROIs is described in the supplementary material and illustrated in Supplementary Fig. 2).

**Figure 1:**
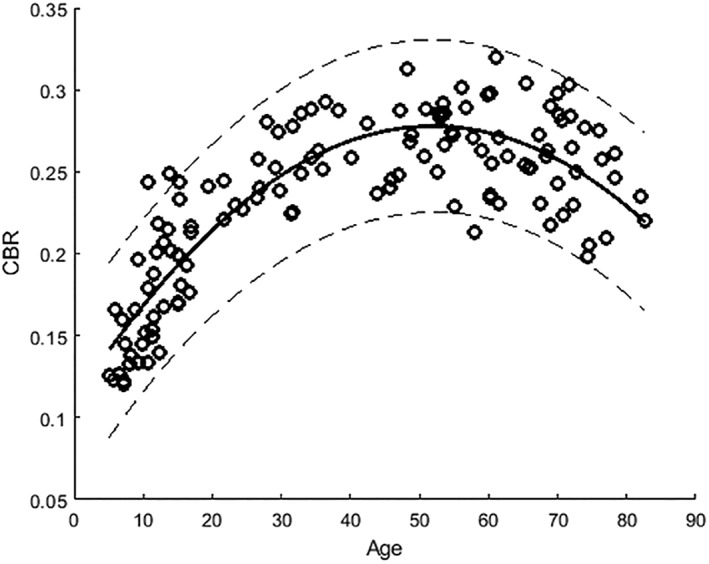
SN contrast‐to‐background noise ratio versus age (open circles) in normal healthy participants (dashed lines represent 95% confidence intervals).

**Figure 2:**
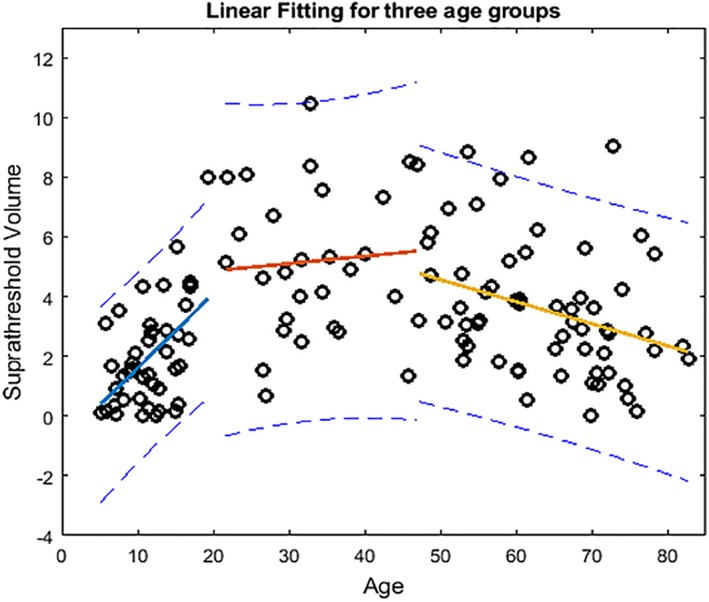
Linear age effects on the normalized suprathreshold NM volume in the 3 age subgroups (for the sake of illustration, demonstrated values in *Y* axis are the real normalized suprathreshold volume multiplied by 100).

To calculate the CBR of bilateral SNs, 2 ROIs were semiautomatically placed in the visually brightest‐appearing anterior and posterior SNs on each side on 3 consecutive slices. Specifically, once the center of the region of interest (ROI) was manually determined, this allowed for automatic placement of 2 circles of 3‐mm radius in the anterior and posterior parts of the hyperintensity area of the left and right SNs, respectively (inset of Supplementary Fig. 2). Using the same approach, 2 control ROIs (5‐mm radius circles) were chosen to define the background signal in the area of cerebral peduncles. We then derived the mean CBR of the left and right SNs, and the anterior and posterior SNs using the mean intensity of each ROI in the SN and the average intensity of the 2 control background ROIs. We measured the CBR using the following equation:

$$\text{CBR}=\left({\mathrm{SI}}_{\mathrm{SN}}{‐\mathrm{SI}}_{\mathrm{BG}}\right){/\mathrm{SI}}_{\mathrm{BG}},$$

where SI_SN_ is the average signal intensity of semiautomatically defined ROIs in the SN and SI_BG_ as the mean background signal intensity of the 2 background ROIs chosen in the cerebral peduncles.

To compute hyperintense SN volumes, we followed the approach described in reference 17 to select an optimized calibration factor (Cal_opt_) accounting for the protocol‐specific signal intensity and contrast variation. The study‐specific calibration factor was found through step‐wise variation of a multiplier (between 1 and 8.25, 0.25 increments) of the individual background (BG) noise term expressed as SD of the signal intensity (SI) of the background region. Derived mean NM volumes for the adult group's mean only were compared with expected pigmented NM volume based on a recent histologic study of subjects with a similar age range (ie, 172.8 ± 34.1 mm^3^^,^ 22; see supplementary material and Supplementary Fig. 2 for details). Individual thresholds were then determined as

$${\mathrm{SI}}_{\text{thre}}={\mathrm{SI}}_{\mathrm{BG}}{}_{\text{mean}}+({\text{Cal}}_{\text{opt}\text{.}}{\times \mathrm{SD}}_{\mathrm{BG}}),$$

where SI_BGmean_ is the mean background signal intensity of the 2 background ROIs (and the same average signal of the background ROIs in each slice obtained for the calculation of CBR) and SD_BG_ is the standard deviation of the background signal intensity.

Examples from different age groups are illustrated in Supplementary Figure 3. We then visually inspected all segmented volumes (blinded to age and sex) to confirm that suprathreshold voxels were within the boundary of the SN. The boundary had to be redefined if structures other than the SN were included. Hyperintense SN volumes were then calculated as the number of validated suprathreshold voxels across 3 slides multiplied by the voxel size. Finally, to control for the potential impact of developmental and involutional changes in brain and midbrain volume, we normalized the hyperintense SN volume to the midbrain volume using another in‐house Matlab script that enabled freehand drawing of the boundary of the midbrain area over the 3 selected slides (examples in Supplementary Fig. 3).

**Figure 3:**
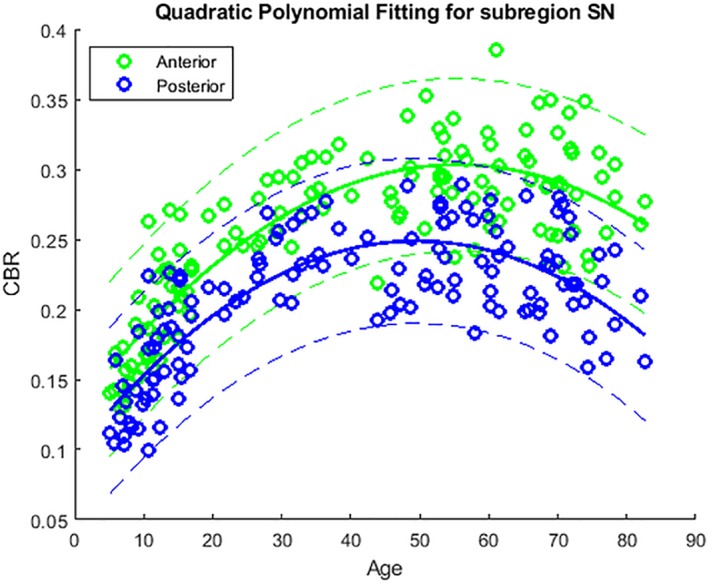
Contrast‐to‐noise ratio of the anterior and posterior SN in healthy participants. Similar relationship patterns of CBR‐SN anterior (green open circles) and CBR‐SN posterior (blue open circles) and age (green and blue curves) are shown. Dashed lines represent 95% confidence intervals.

Based on visual inspection of the age‐dependence of the NM signal, we divided the population into 3 age groups (<20, 20‐47, and >47 years) for post hoc analyses. We then tested for age associations within these 3 subgroups using linear regression and group comparisons to quantify changes of SN volume and brightness during development, adulthood, and middle‐old age.

Half of both the volumetric and the CBR values were randomly selected and repeated by the same viewer (S.A., 2 years' experience) and a second viewer blindly (Y.X., 8 years of experience in brain‐imaging research) to derive the intraclass and interclass reliability. Intrarater reliability of midbrain volumes was assessed in three‐quarters of the cases, and intraclass reliability was examined. Statistical tests were performed using IBM‐SPSS for Windows (version 21.0; IBM, Armonk, NY).

For the main research question on the type of age dependence of the NM signal, we used linear and nonlinear regression analyses for volume and contrast ratios. The *R* ^2^ values of these regression analyses after adjusting for the number of variables in the model (adjusted *R* ^2^) were compared to assess which regression equation best described the relationship between age and NM content in SNpc.

For comparison of NM volume and contrast ratios between sexes, hemispheres (left vs right), and subregional differences of CBR in SN (anterior vs posterior), we conducted univariate or multivariate analyses of variance with least‐squares differences post hoc comparison or *t* tests.

Because sex was not completely matched in all age groups (Supplementary Fig. 1), we also compared (post hoc) NM volume and CBR between men and women in each of the 3 age groups. Further post hoc tests were undertaken based on visually defined age subgroups and to confirm putative threshold dependence for the young cohort. Where data were not normally distributed, we applied nonparametric tests. Significance was defined at α = 0.05. Values are given as mean ± standard deviation unless stated otherwise. Intraclass and interclass correlation coefficient analyses were performed.

## Results

Data from 5 participants (∼4%, including 3 children and 2 adults > 65 years old) had to be excluded because of movement artifacts after visual inspection. The final analysis thus included 129 healthy participants aged 5.1 to 82.7 years (mean age ± SD, 40.5 ± 24.3 years; 56 males and 73 females). Figure 1 in the supplementary material illustrates the distribution of age and sex of our cohort. The intraclass and interclass correlation coefficients for intrarater and interrater concordance of SNpc suprathreshold volumes were 0.921 (95% CI, 0.867‐0.953) and 0.913 (95% CI, 0.854‐0.948), and those of SN‐CBR were 0.985 (95% CI, 0.975‐0.991) and 0.937 (95% CI, 0.895‐0.963). The intraclass correlation coefficient for midbrain volumes was also very high (0.966; 95% CI, 0.835‐0.987).

### Age Effects of the Contrast‐to‐Background Ratio of the Pigmented Substantial Nigra and Its Subregions

Visual inspection (Fig. 1) shows a rapid increase in the CBR of SNpc in those up to 20 years of age, with a slow increase and a plateau between about 45 and 53 years of age, followed by a decrease with increasing age. We also observed increased intersubject variability with older age.

All the nonlinear fitting approaches showed significant correlations between age and SN‐CBR (*P* < 0.0000001); see Supplementary Figure 4. However, the quadratic regression demonstrated the simplest fitting curve with a high adjusted *R* ^2^ (0.81). The equation was as follows: Y = ‐0.0001*X* ^2^ + 0.0065*X* + 0.1101 (Fig. 1). Life span trajectories of NM contrast changes were similar in both the anterior and posterior SNs, with an adjusted *R* ^2^ of 0.82 and 0.74 (*P* < 0.0000001).

*Post hoc tests*: Based on the qualitative assessment, we further assessed the age effects across and within the 3 formed subgroups (see supplementary material).

### Age Effect on the Pigmented SN Volume

The qualitative assessment suggests a similar but more dispersed age trajectory for pigmented SN volumes compared with those we found for SN CBR (Fig. 1).

Again, on the basis of the qualitative assessment, we examined the age effect for 3 subgroups. Our results showed there was a significant age effect (1‐way analysis of variance) across the 3 age groups (Table 1 summarizes the mean ± SD of each group and the significance of their comparisons) showing that the normalized pigmented SN volume in the 20‐ to 47‐year group was significantly higher than those >47 years, as well as those in the youngest group old). Also, there was a difference in pigmented SN volume in the >47‐years‐old group versus the <20‐years‐old group. Figure 2 illustrates our findings of significant linear age effects on the normalized suprathreshold NM volume in children to adolescents < 20 years old (*R* = 0.50; *P* = 0.0011) and also significant, but reversed age effects in those > 47 years old (*R* = 0.28; *P* = 0.031), with data fitted by 2 lines, *Y* = 0.0041*X* ‐ 0.0108 and *Y* = ‐0.0007*X* + 0.0782, controlling for sex. From 47 years old, we found a 5.3% loss per decade of life, calculated from CBR values averaged over each decade, and 3.9% loss when estimated using our CBR linear fitting. No significant age effects were seen between 20 and 47 years old (*P* = 0.38).

**Table 1:** Summary of pigmented SN related metrics for the three age subgroups

Age group (total:female)	<20 (41:22)	20‐47 (27:14)	>47 (61:38)	One‐way ANOVA	Pairwise comparisons (*P*)	
		Value × 100 (Mean ± SD)		<20 vs 20‐47	<20 vs > 47	20‐47 vs > 47
Normalized volume	2.0 ± 1.8	5.2 ± 2.5	3.5 ± 2.2^a^	< 0.000001	< 0.0001	0.004
CBR total–SNpc	17.5 ± 3.8	25.4 ± 2.2	26.3 ± 2.9^a^	< 0.000001	< 0.000001	0.09
CBR Ant–SNpc	19.2 ± 4.0	27.3 ± 2.5	29.4 ± 3.3^a^	< 0.000001	< 0.000001	0.0064
CBR post–SNpc	15.9 ± 3.8	23.3 ± 2.5	23.0 ± 3.4^a^	< 0.000001	< 0.000001	0.8193

^a^significant group difference, *p* < 0.000001.

### Sex, Laterality, and Subregional Effects on Substantia Nigra Pigmentation

The normalized volume of the pigmented SN in females was significantly larger than in males in the oldest age group (males, 0.027 ± 0.022; females, 0.039 ± 0.024; *P* = 0.024). No other difference in volume or CBR was found between the sexes (*P* = 0.79).

In addition, no laterality effect was found for either normalized SN volume or CBR. The left pigmented normalized SN volume (0.016 ± 0.012 mm^3^) was similar to the right SNpc (0.018 ± 0.013 mm^3^); *P* = 0.406. The CBR was also bilaterally similar (left SNpc, 0.231 ± 0.052; right SNpc, 0.236 ± 0.053; *P* = 0.293). However, across the life span, the anterior SN (0.253 ± 0.057) displayed higher CBR than the posterior SN (0.209 ± 0.048); *P* < 0.0000001 (Fig. 3). We further assessed any coil inhomogeneities that might affect the laterality effect but found no significant differences when comparing the mean intensities within the left and right background ROIs (*P* > 0.05).

## Discussion

NM‐sensitive MRI in a large, comprehensive life span sample of healthy volunteers demonstrated that SN pigmentation steeply increases from early childhood to young adulthood, plateaus in middle age, and declines in the sixth decade following an inverted U‐shaped trajectory. Brightness was best described by a quadratic fit, with contrast ratios yielding a significantly better fit than volume, explaining 81.5% of the variance. Regional differences were found, with brighter anterior versus posterior SN across all ages.

### Neuromelanin Strongly Increases in Childhood and Young Adulthood

Using an optimized NM‐sensitive MRI and custom scan preparation (https://www.youtube.com/watch?v=IMpfW8KtoE8),^28^ we visualized the NM‐rich region in the SN in vivo in young children from 5 years as the earliest age when sedation‐free MRI can be well tolerated. We were able to estimate NM CBR and pigmented NM volumes using a dedicated brief NM‐sensitive sequence with an age‐adjusted threshold. We found a steep increase in both brightness and volume during childhood‐adolescence. Thus, our in vivo findings are well in line with histological reports that NM deposition appears to start at birth or at a very early stage of life,^19^, ^20^, ^29^, ^30^ with an almost linear increase of NM‐containing neurons in the first 2 to 3 decades of life. Linear fits explained > 71% of NM contrast variance and about 50% of the variance of NM volume in our cohort, suggesting a stronger developmental effect on the degree of pigmentation than the volume of pigmented SNpc. Our findings also agree well with earlier studies showing that the degree of pigmentation of the SN is greater in the adult brain than in children and adolescents.^29^, ^30^

NM is an insoluble complex of pigment, lipid, and protein found in cytosolic organelles of catecholaminergic neurons that do not undergo physiological degradation, thereby explaining its accumulation. In fact, NM was proposed to reflect a chronological metric of cumulative metabolic waste.^20^ Biosynthesis of the melanic component is directly linked to catecholaminergic metabolism, and in the SN it is specifically associated with oxidation of nonvesicular dopamine.^1^

### Neuromelanin Declines From Middle to Old Age

In line with the chronometric hypothesis of NM synthesis, a continued linear signal increase would be expected and has been suggested.^31^ In contrast, our current study clearly demonstrates that MRI detected nigral NM brightness and that volume changes little during adulthood and decay from about the mid‐5th decade in healthy aging. The observed age trajectory of the SNpc NM contrast shows a remarkable similarity with the largest available life span postmortem sample,^32^ as further validation that neuromelanin content explains the observed MRI contrast. A similar quadratic age trajectory, although only explaining less than 15% of NM contrast, was reported in the LC in 64 healthy subjects spanning adulthood to old age.^26^ The weaker effect may be because of partial volume effects of the smaller LC, less pigmentation, and different levels of metals and other chemicals.^33^ Nevertheless, the similarity of the trajectory of signal changes with signal reduction observed in both the LC^26^ and the SN in our study suggests an involutional loss of NM pigment in catecholaminergic nuclei in old age. Other histological studies demonstrated that the number of pigmented SN neurons declines significantly, ranging from 28.3% to 66.7% while aging from ∼20 to 90 years old.^21^, ^22^, ^32^, ^34^ The discrepant observation of the increase in nigral NM pigmentation throughout adult life in 1 series of postmortem samples^31^, ^35^ might be explained by cohort differences, differences in NM detection methods (as argued by reference 35), and possible conformational pigment changes during aging.^4^ Using our CBR measures, we estimated ∼4.6% loss per decade of life from 47 years old over our decade groups, which is consistent with the loss rate reported in other works.^21^, ^22^

MRI appearances of preferential contrast over volume reduction of pigmented SN in old age could be explained by either NM loss or reduced NM iron content. The latter is unlikely, as total brain iron and also SN iron are known to increase with age,^24^, ^36^ and there is also no evidence for reduced iron‐chelating capacity of NM in old age. Future work is needed to elucidate how the change of free iron together with reduced ferritin‐NM compound translate into the NM‐ and iron‐sensitive images.^37^ Taken together the observed age effect of NM decline from middle age is most likely a marker of degeneration of dopaminergic neurons, thereby offering a window for future studies into age‐related brain stem degeneration and its link to PD pathogenesis.

### Neuromelanin, Iron, and Vulnerability to Neurodegenerative Disorders

Previous evidence points to an important role of iron in the normal development of the dopaminergic system and degeneration.^33^, ^38^, ^39^ Age‐related increase in the concentration of brain iron was found to be related to increased blood‐brain barrier permeability, neuroinflammation, and redistribution of iron.^40^, ^41^ The synthesis of NM helps the sequestration of toxic metals, such as iron overload, which could exert a neuroprotective effect.^2^, ^24^, ^42^, ^43^, ^44^ Conversely, extracellular NM may be involved in the pathogenesis of PD by triggering neurodegeneration via an immune‐based pathogenic mechanism.^2^, ^45^, ^46^, ^47^ The subregional distribution difference of NM‐bound iron and its possible divergent age effects might offer an explanation for the link between NM, iron, and age‐related neurodegeneration of brain stem nuclei.^38^, ^48^, ^49^, ^50^ The iron deposition in SN in PD has been investigated using different MRI techniques. However, the findings are not consistent.^51^, ^52^, ^53^, ^54^, ^55^, ^56^, ^57^ This discrepancy may be ascribed to the incongruence of the definition of SN based on different MRI approaches (see review in reference 58). Recent studies analyzed the iron level in the SN defined by NM MRI, and they have consistently demonstrated increased T2*‐weighted hypointensity in lateral‐ventral SNc in patients. This loss of hyperintensity in the nigrosome‐1, which resembles the swallowtail, was proposed to reflect iron deposition and neuromelanin loss underlying the pathology of Parkinson's disease.^59^ Our findings of an anterior‐posterior gradient in SNpc contrast with lower CBR in the posterior SN also echoes this previous imaging and pathological evidence.^17^, ^21^, ^51^ Importantly, we found that the NM gradient was present from an early age and persisted over the life span, suggesting an age‐independent regional effect that may confer resilience to PD pathology^35^ and thus support an NM neuroprotective role. It is noticeable that we also found bigger normalized SN volumes in women compared with men older than 47 years, which may underpin the high male‐to‐female ratio of the prevalence of PD.

Our findings are broadly in line with a putative neuroprotective role of NM, but longitudinal in vivo NM MRI in combination with iron mapping will be needed to fully elucidate the role of subregional NM and iron content during healthy aging and early neurodegeneration. An increasing body of imaging evidence supports the value of NM MRI as a disease biomarker in Parkinson's disease. Our previous results and other studies using NM MRI have shown a significant decrease of neuromelanin‐related signals and/or volume in SN in PD, allowing for higher discrimination accuracy between PD and age‐matched healthy controls.^15^, ^16^ Evidence also demonstrated that volume loss correlated with disease duration and severity.^17^ Furthermore, preliminary longitudinal findings suggested that neuromelanin MRI might be used as an indicator of disease progression.^10^ A very recent review recommends that NM MRI may be useful for regularly screening older participants at risk for PD and for detecting PD at the preclinical stage.^60^ Our study now provides normative findings across a wide age span that will facilitate the study of the role of NM as a risk factor of and in presymptomatic PD and as a mechanistic outcome marker in future NM and iron‐targeted interventions.

### Strength, Implications, and Limitations

Our study used an optimized brief (less than 4 minutes) MRI protocol for noninvasive in vivo mapping of neuromelanin‐sensitive signals. This is the first comprehensive life span study of the NM MR signal in healthy subjects in vivo. There are a few limitations to this retrospective and cross‐sectional MRI study. We cannot exclude the possibility of some of the older participants having presymptomatic neurodegenerative diseases, and only 66.7% of those > 47 years old underwent cognitive testing and clinical screening, whereas others self‐reported their health and cognition status.

## Conclusions

We describe a strong nonlinear age effect on brain stem pigmentation, with an inverted U‐shaped association providing the first detailed trajectory of MR‐detectable brain stem pigmentation and depigmentation from childhood to old age. Our fast protocol makes it possible to investigate the role of NM in brain development and to longitudinally assess risk and moderator factors of physiological and pathological brain stem depigmentation, shedding light on the putative neuroprotective and neurotoxic effects of NM and iron.

## Supporting information

Supplementary Material
