# Supplementary material for: Life span pigmentation changes of the substantia nigra detected by neuromelanin‐sensitive MRI
Source: Mov Disord. 2018 Nov 13;33(11):1792–9. doi: 10.1002/mds.27502 (PMC6659388; doi:10.1002/mds.27502)
Supplement: Supplementary file 1 — Supplementary Material [file MDS-33-1792-s001.docx]

**Supplementary material:**

*Research ethics:*

The projects which contributed data to this analysis, with details of the chief investigator (CI), funding source and Research Ethics Committee (REC) reference, are as follows: The Parkinson's MR Imaging Repository (PAMIR), CI Prof DP Auer, funded by Parkinson's UK, NHS REC 14/EM/0061; Brain Network Dysfucntion in Multiple Sclerosis (BRaNDy-MS), CI Dr. RA Dineen, funded by the MS Society, REC 14-EM-0064. The Childhood Ataxia Telangiectasia Neuroimaging Assessment Project (CATNAP), CI Dr. RA Dineen, jointly funded by The A-T Children's Project and Action for A-T, NHS REC 14/EM/1175; The Normal Variability Evaluation for MRI in children and young people (NOVEL-MRI) study, CI Dr. RA Dineen, funded by The A-T Children's Project, University of Nottingham Medical School REC L14112016; and the Development and Optimisation of Novel MRI Techniques study, CI Dr RA Dineen, University of Nottingham Medical School REC B12012012a.

*Inclusion and exclusion criteria:*

For the children and young people recruited through the projects NOVEL and CATNAP, potential participants were excluded as per protocol if they had a 'history of the neurological or neurosurgical disorder, or other significant medical histories'. This was ascertained by checking with the parent/carer, and directly with the participant (if 16 years or older), at the time of the pre-recruitment screening. No additional examinations or investigations were conducted.

For the adults, we included participants who were initially recruited for the PaMIR study who had to 1. Be of good general health with no significant past medical history; 2. Be able and have the capacity to give informed consent; 3. Have very good comprehension of written English and be fluent in English, in order to complete the cognitive (mini-mental state examination) and depression (Beck's depression inventory) tests. Participants were excluded if they 1. Had any contraindication to Magnetic Resonance Imaging, such as claustrophobia or metal implant; 2. were pregnant and regarding potential pregnancy, participants might be asked to undergo a pregnancy test; 3. Had a diagnosis of any major neurological, neurosurgical or psychiatric condition; or 4. Were currently taking medications known to have a dopaminergic effect. We additionally recruited healthy volunteers from the BRaNDy-MS project to achieve a representative age distribution using similar inclusion / exclusion criteria without undergoing cognitive or depression tests.

In our Age>60 group, 84.2% of our participants underwent cognitive tests and depression scoring and were all within the normal range [1-2]. In our Age>47 group, 66.7% subjects underwent both cognitive and depression tests and their scores were all within the published normal range [1-2]. All investigations with questionnaires, medical history taking and assessment of medication history were undertaken by Dr. Naidu, a clinical research fellow in Neurology.

**
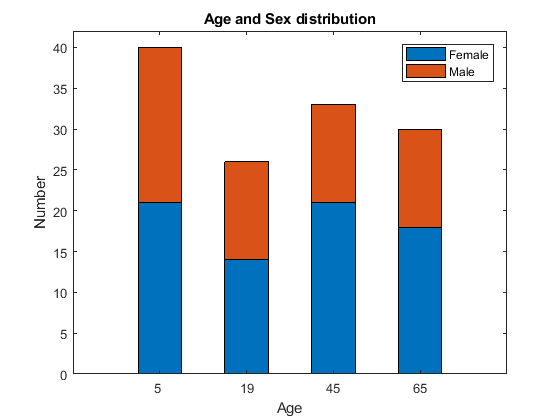
**

Figure 1: The distribution of the age of our cohort. Stacked bar represents the total number of participants and the blue segments represent the number of male participants.

The aim of the threshold adaptation is to calibrate the protocol-dependent variation of signal intensities of both the neuromelanin rich and background regions. To achieve this, but maintain inter-individual variation the group’s average, neuromelanin rich SN volume is approximated to the published volumes. For this study comprising a different age group as in [3] we chose a recent reference histologic study comprising an age 18-90 years cohort, which reported that the SN is composed of approximately 68% pigmented neurons, equivalent to 172.8 ±34.1 mm3 in volume [4].

During the optimisation of the threshold for this study protocol and its adult cohort, we then estimated the groups’ mean suprathreshold volume in a step-wise approach using a range of multipliers (1 to 8.25 in 0.25 increment). Based on these multipliers, we computed the individual threshold as SI threshold = BG mean + (range of multipliers × SD BG) whereby BG mean and SD BG were individually determined.

We used a multiplier of 6 to determine the individual thresholds that best suited to approximate the group's average SN volume for this study population. The threshold was also suited to the protocol to the expected pigmented volume of SN in an age-matched histologic study [4] (the vertical line in Supplementary material Figure 2).


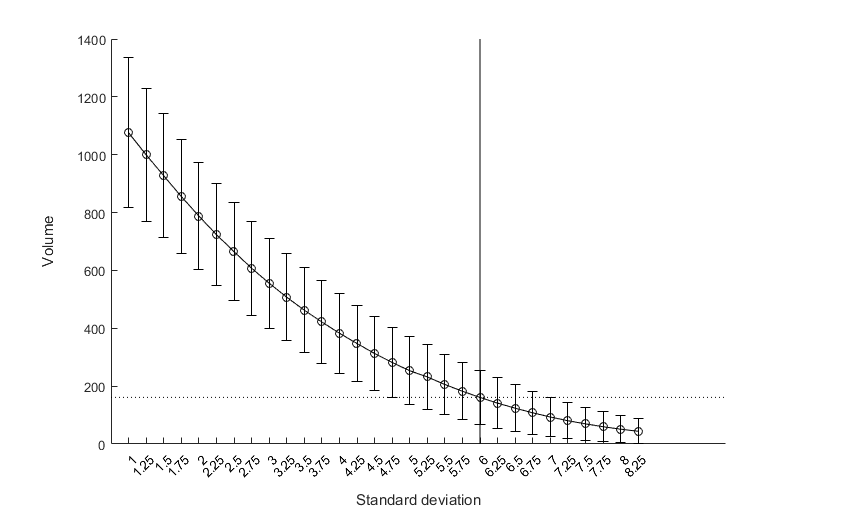


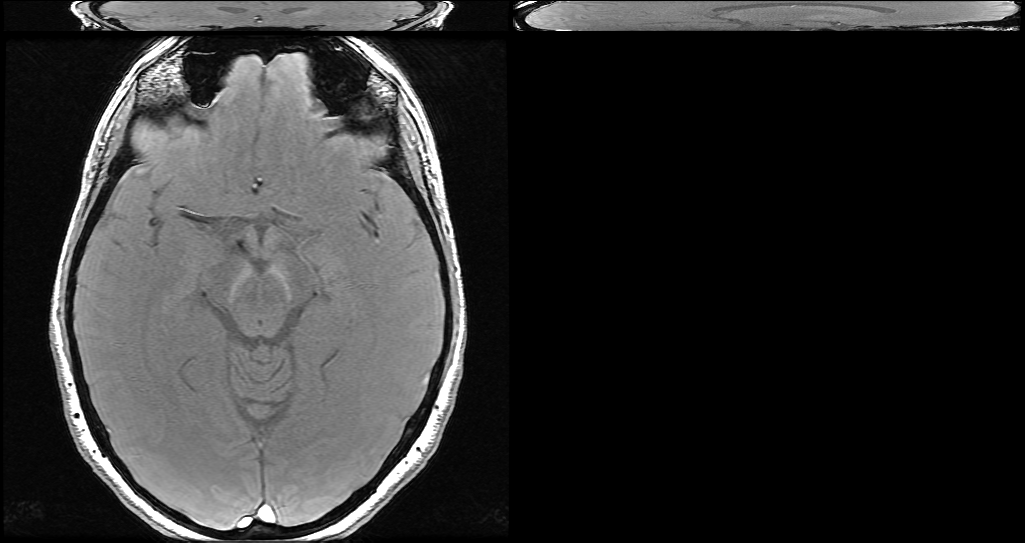


Figure 2: Definition of the background and SN regions-of-interest and the threshold used for the calculation of the suprathreshold volume of neuromelanin related signal intensity. The graph indicates that using 6 SDs above mean background signal intensity matches the best of the estimated pigmented volume of SN showed in an age-matched histologic study.


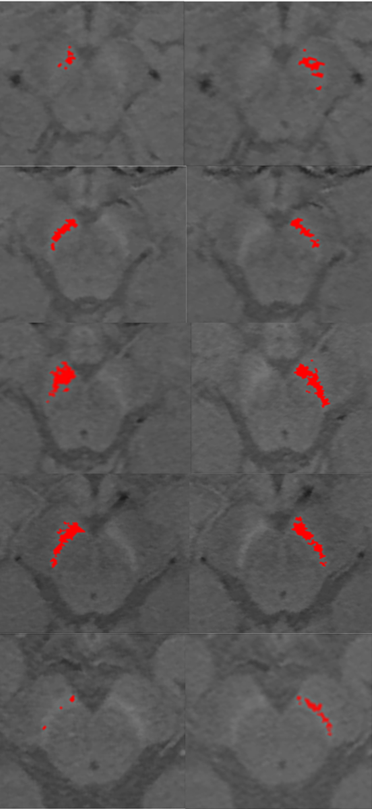


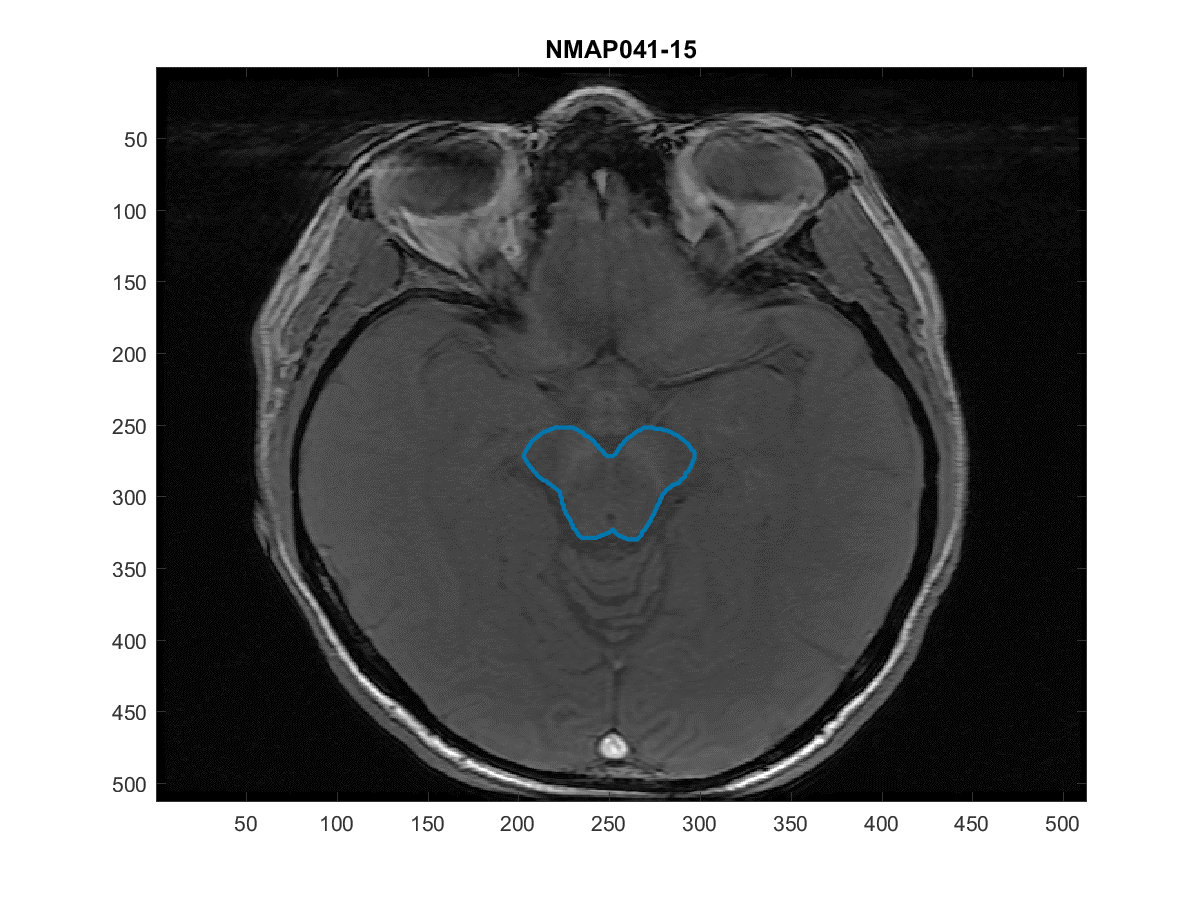

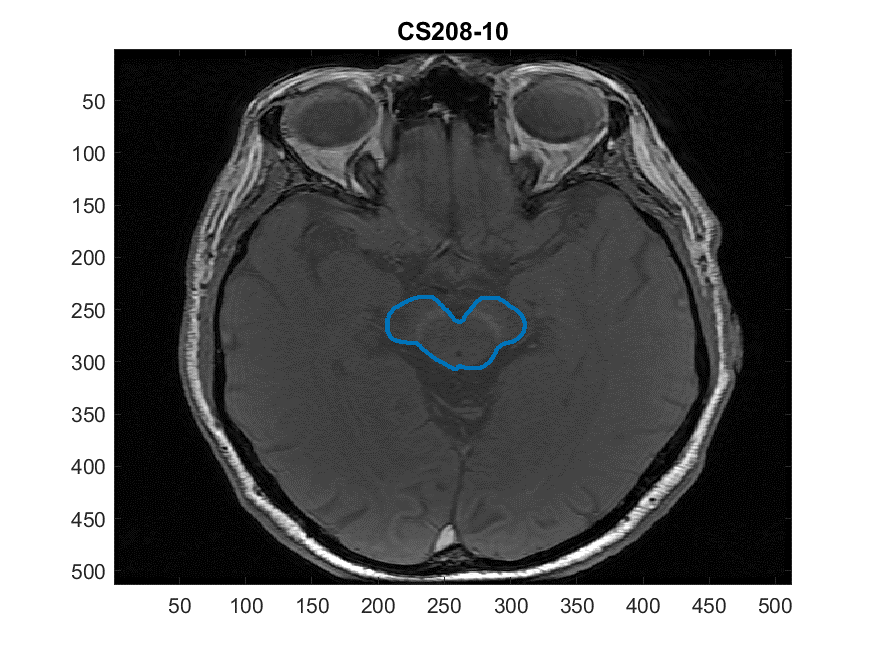

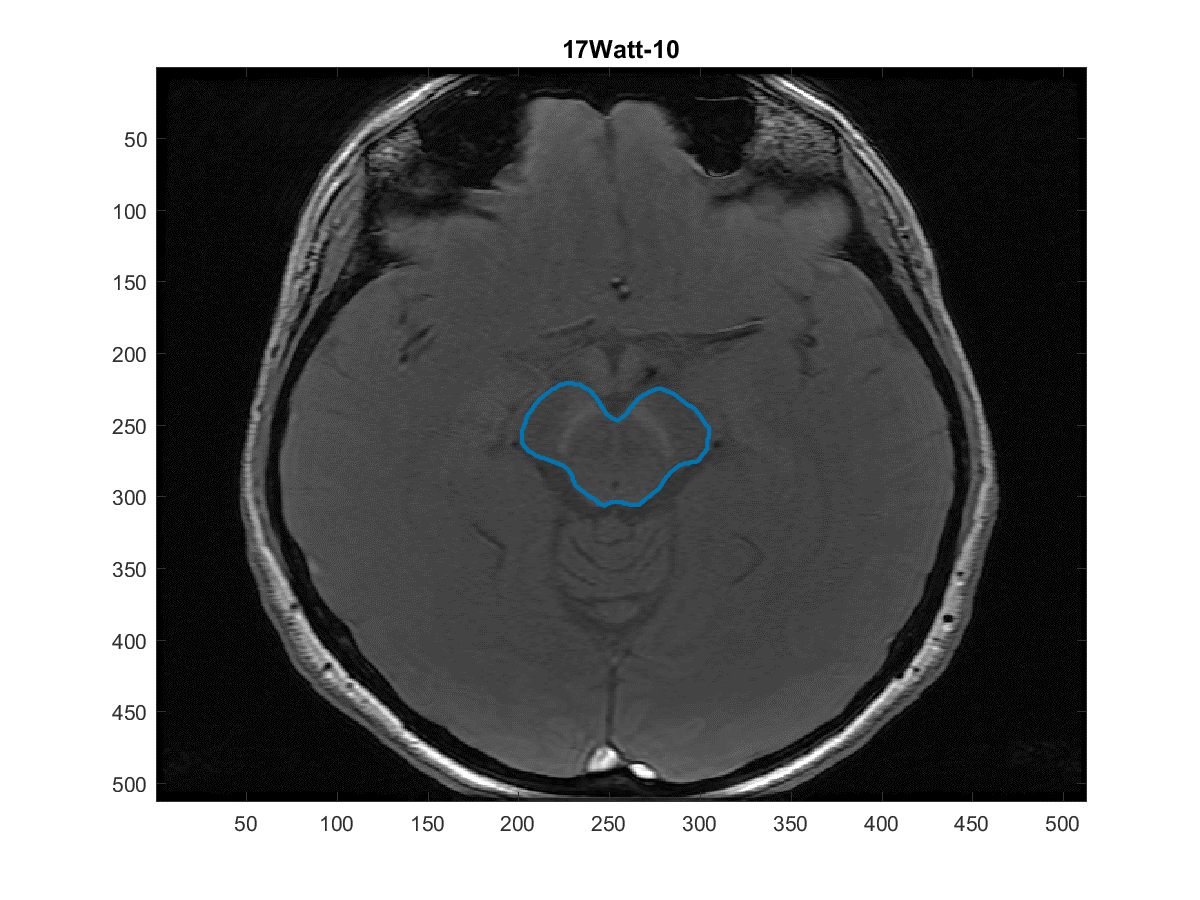

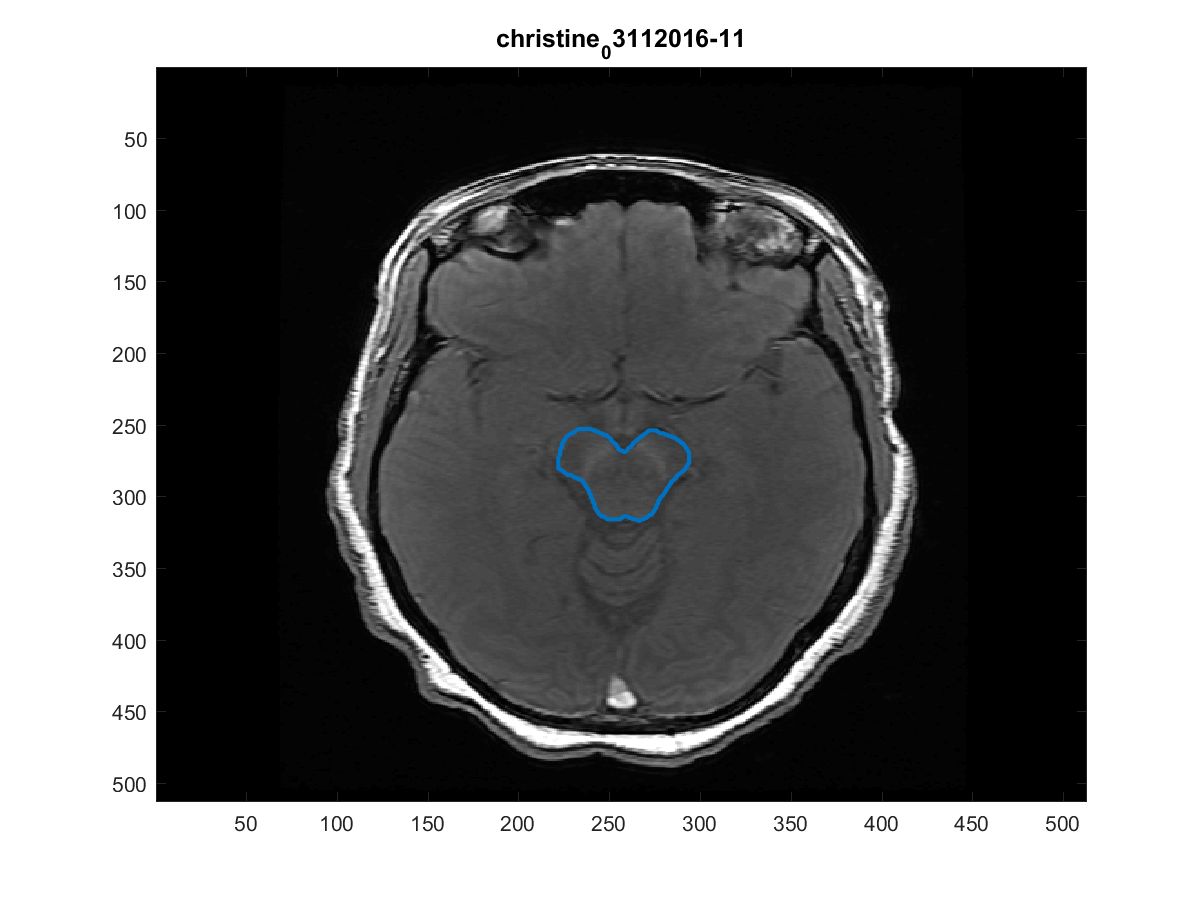

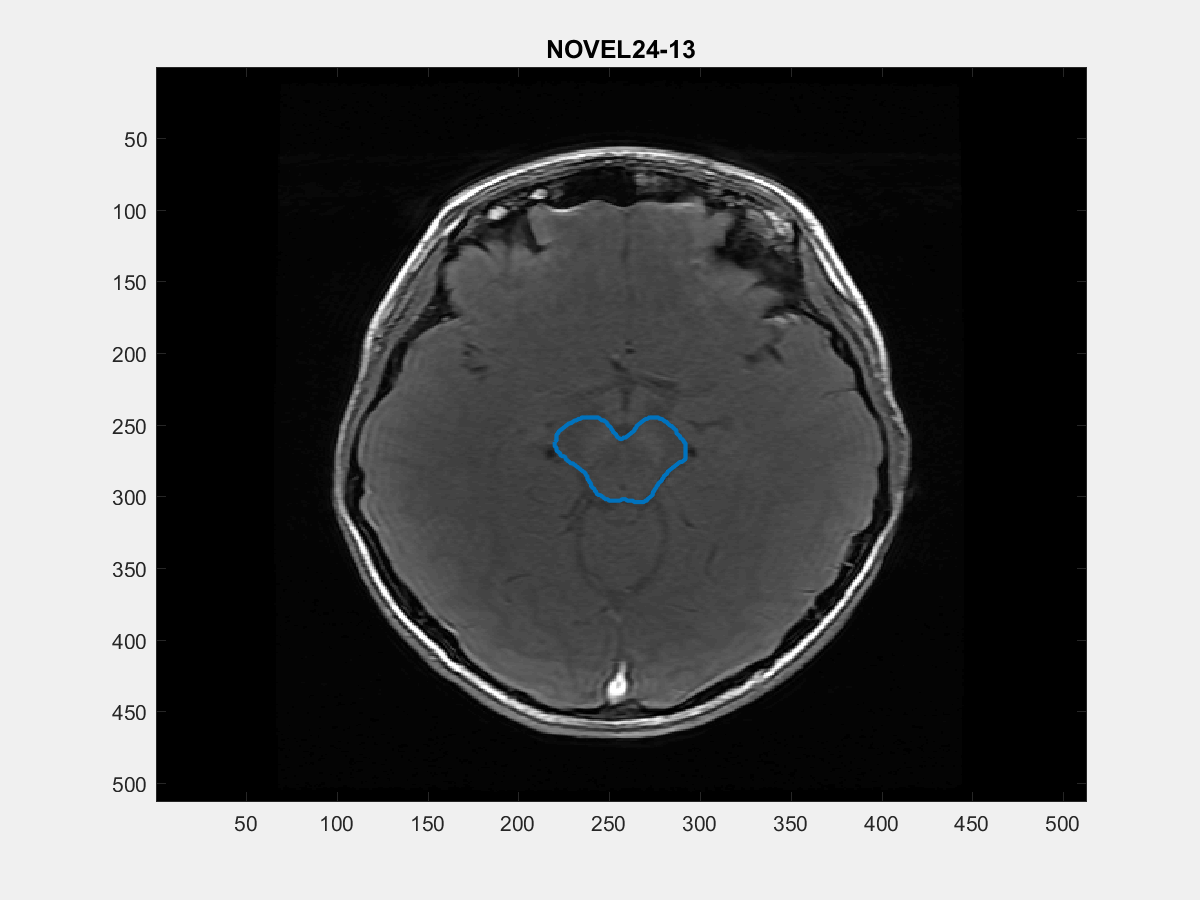


Figure 3: Exemplars of the remaining voxels (red regions) in the middle slice after applying the threshold to different participants and the delineation of the midbrain boundary of those slices in the same participants. Top to bottom for suprathreshold voxels and left to right midbrain boundary: 8.91-year-old male, 27.8-year-old female, 46.0-year-old female, 61.2-year-old female and 74.4-year-old male.


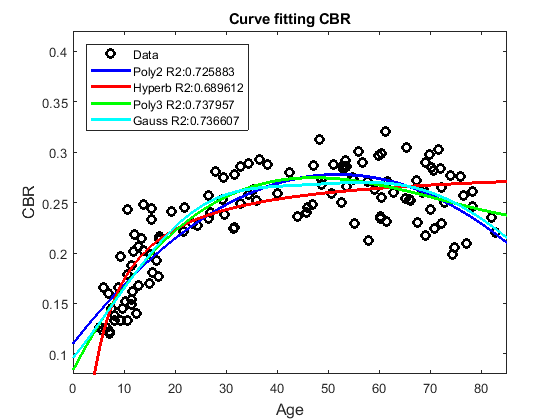

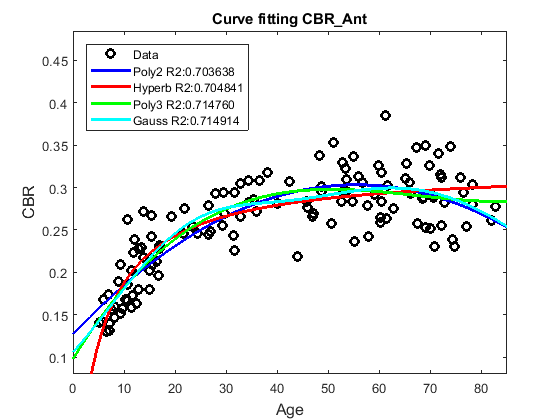


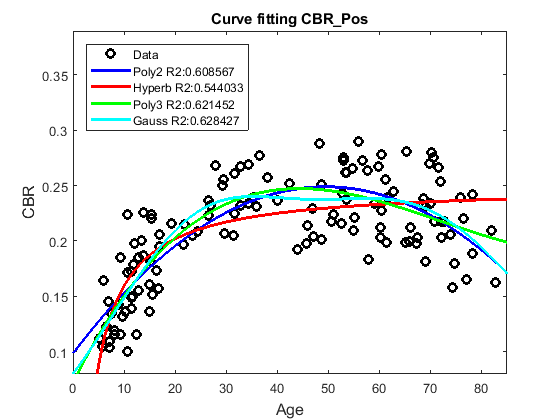


Figure 4 A: The solid curves in different colors represent different nonlinear fittings for SN contrast to noise ratio vs age, B: for CBR-SN anterior and C: for CBR-SN posterior.

Post-hoc tests results for the three subgroups of CBR:

Our results showed that there was a significant age effect (one-way ANOVA) across the three age groups (p<0.0000001) (suppl material Figure 5 and table below) with significantly lower contrast ratio in the <20 years age vs 20-47 (p<0.0000001), and remained significantly lower than those in the oldest >47 years group (p<0.0000001). No difference of pigmented SN brightness in the >47 years group vs 20-47 group (P=0.09) was found. However, when we further divided the >47 age group into 47~60 and >60, the >60 age group showed significantly lower CBR compared to those in 47~60 age group (P= 0.0048). In addition, by fitting the age-CBR within the three subgroups, we found significant linear age effects on the contrast ratio in both children to adolescents <20 (R=0.71; P<0.0000001), and 20 ~ 47 years (R=0.483, P=0.0167) and also significant, but reversed age effects in those >47 (R=0.41; P=0.0016) with data fitted by three Y= 0.0075X+0.0888, Y=0.0017X+0.2001, and Y=-0.0012X+0.335 controlling for sex. Although no difference was observed between 20-47 vs >47 groups, our post-doc tests altogether suggest that following a rapid increase in the CBR of SNpc up to 20 years of age with less steep increase up until 40-50 years of age, there was a decrease of CBR with increasing age, which is consistent with the findings reflected in our quadratic curve (Figure 2 in the main text).


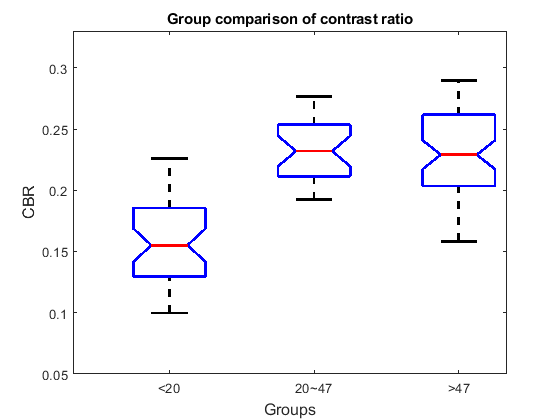

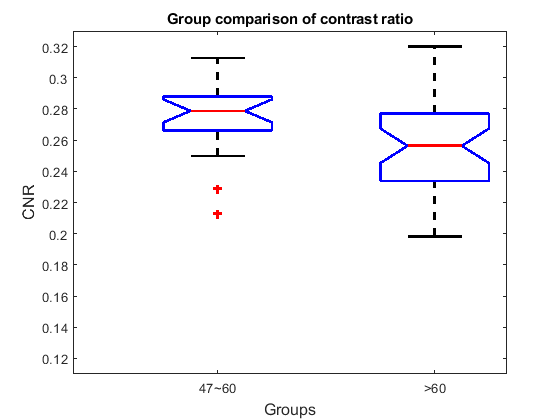


Figure 5: Left: The CBR of SNpc in three age groups illustrated using notched box plots. Right: Further divided the >47 age group into 47~60 and >60 and >60 showed a significant decrease compared to 47~60 age group (P= 0.0048). Upper whiskers and lower whiskers: maximum and minimum value; upper and lower boundary of the bar: 75^th^ and 25^th^ percentile; short red vertical line: median; the notch: 95% confidence interval of the median; +: possible outliers.


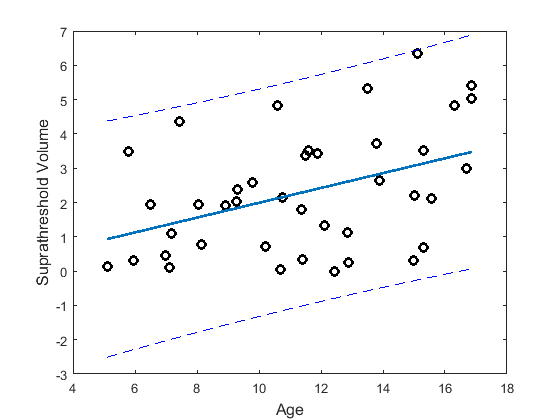


Figure 6: We then also explored additional (5.75 instead of 6 as the multiplier, based on visual inspection only) thresholds for participants <18 for a confirmatory analysis in the young subgroup alone. This was to investigate whether our main findings are subject to a floor effect in the younger cohort as we observed that a few participants <18 showed near-zero volumes. Importantly, when lowering the threshold for the population under 18, the main findings were of age dependency of the NM volume were not affected. This figure is a linear fit of age-normalised NM volume for those <18 age thresholded with a lower threshold.

References:

[1] Leentjens AF et al., Mov Disord. 2000 Nov;15(6):1221-4.

[2] W.A. Kukull et al., Journal of Clinical Epidemiology, Volume 47, Issue 9, 1994, Pages 1061-1067

[3] Schwarz, S.T., et al., In Vivo Assessment of Brainstem Depigmentation in Parkinson Disease: Potential as a Severity Marker for Multicenter Studies. Radiology, 2016: p. 160662.

[4] Ma, Röytt, Collan, and Rinne, (1999), Unbiased morphometrical measurements show loss of pigmented nigral neurones with ageing. Neuropathology and Applied Neurobiology, 25: 394-399. doi:10.1046/j.1365-2990.1999.00202.x
